# Primary open-angle glaucoma: Reader feedback

**Published:** 2013

Thank you to everyone who commented on our double issue on primary open-angle glaucoma (Comm Eye Health J 2012; 25(79&80).

**John Sandford-Smith (UK)** pointed out both an error and an omission. In ‘Detecting possible glaucoma with only limited equipment’, the last sentence of section 6 (page 49) reads: ‘Remember. A RAPD is not only caused by a cataract.’ In fact, a cataract in an otherwise healthy eye **never** causes a RAPD. Apologies both to the authors and our readers for the error, which occurred during editing. The ommission was in the panel about sub-conjunctival 5FU injection, on page 75, where no advice was given about dose strength and volume. In response, the article's author suggests a dose of 10 mg. If using 5FU at a concentration of 50 mg/ml, the volume injected would be 0.2 ml.

**Daniel Laroche (USA)** disagreed with the statement that IOP below 21 is ‘normal’: ‘There is good evidence that the mean normal IOP is 15 and the mean glaucoma IOP is 18. When I see patients with IOP of 17–19 I look carefully at their nerve fibre layer to ensure there is no early glaucoma.

Finally, **Hugh Taylor (Australia)** suggested that more emphasis should be placed on the the family history of glaucoma: “A family history of glaucoma will increase the chances of developing glaucoma up to eight times. This has a far bigger impact than any other known risk factor. We have to work hard to make sure that all patients with glaucoma inform their relatives. When we are examining patients in general, we must also specifically ask them about their family history.”

The letters are available in full on www.cehjournal.org

